# B-Cell Acute Lymphoblastic Leukemia in a Child with Down Syndrome and High-Risk Genomic Lesions

**DOI:** 10.3390/cimb47090704

**Published:** 2025-09-01

**Authors:** Cristina-Crenguţa Albu, Florin Bica, Laura Nan, Lucia Bubulac, Claudia Florina Bogdan-Andreescu, Ionuţ Vlad Şerbanică, Cristian-Viorel Poalelungi, Emin Cadar, Andreea-Mariana Bănățeanu, Alexandru Burcea

**Affiliations:** 1 Department of Genetics, Faculty of Dentistry, “Carol Davila” University of Medicine and Pharmacy, 020021 Bucharest, Romania; cristina.albu@umfcd.ro; 2 Department 14, Faculty of Medicine, “Carol Davila” University of Medicine and Pharmacy, 020021 Bucharest, Romania; florin.bica@umfcd.ro; 3 Department of Dentistry, Caraiman Multifunctional Medical Complex, 011561 Bucharest, Romania; laura2nan@gmail.com; 4 Department of Family Medicine, Faculty of Medicine, “Carol Davila” University of Medicine and Pharmacy, 020021 Bucharest, Romania; lucia.bubulac@umfcd.ro; 5 Department of Speciality Disciplines, “Titu Maiorescu” University, 031593 Bucharest, Romania; andreea.banateanu@prof.utm.ro (A.-M.B.); alexandru.burcea@prof.utm.ro (A.B.); 6 Doctoral School, “Carol Davila” University of Medicine and Pharmacy, 020021 Bucharest, Romania; 7 Department 13, Faculty of Medicine, “Carol Davila” University of Medicine and Pharmacy, 020021 Bucharest, Romania; cristian.poalelungi@umfcd.ro; 8 Faculty of Pharmacy, “Ovidius” University, 900470 Constanta, Romania; emin.cadar@365.univ-ovidius.ro

**Keywords:** Down syndrome, acute lymphoblastic leukemia, diagnosis, management, personalized therapy, minimal residual disease, supportive care

## Abstract

**Background:** Acute lymphoblastic leukemia (ALL) is the most common pediatric malignancy, with cure rates exceeding 80% due to advancements in treatment protocols and supportive care. However, in children with Down syndrome (DS), ALL (DS-ALL) presents distinct genomic and clinical challenges. These include mutations in Janus kinase 2 (*JAK2*), neuroblastoma RAS viral oncogene homolog (*NRAS*), and E1A-binding protein p300 (*EP300*), as well as cytokine receptor-like factor 2 (*CRLF2*) rearrangements—such as *P2RY8-CRLF2* fusion—and intrachromosomal amplification of chromosome 21 (*iAMP21*). These aberrations are associated with poor prognosis and increased risk of relapse. The objective of this study was to present a unique DS-ALL case with five concurrent high-risk genomic lesions and to contextualize its management in light of existing literature, emphasizing minimal residual disease (MRD)-guided therapy and supportive care. **Case Report and Results:** We present the case of a three-year-old boy with DS and B-cell ALL (B-ALL), in whom multiple high-risk genomic features co-occurred. Despite these adverse prognostic markers, the patient achieved complete remission following an intensive high-dose induction protocol. We also discuss therapeutic strategies that aim at balancing individualized treatment approaches with optimized supportive care to reduce toxicity and minimize relapse risk. **Conclusions:** This case underlines the importance of comprehensive molecular diagnostics, serial MRD monitoring, and personalized multidisciplinary care in DS-ALL.

## 1. Introduction

Acute lymphoblastic leukemia (ALL) represents the most common malignancy of childhood and remains a continuous priority of pediatric oncology research due to its complex biology and variable clinical outcomes. While advances in molecular diagnostics, risk-adapted chemotherapy, and supportive care have substantially improved survival rates in the general pediatric population, specific subgroups continue to experience poor prognoses. Among these, children with Down syndrome (DS) are particularly vulnerable, exhibiting a markedly increased incidence of ALL and a distinct spectrum of clinical and molecular characteristics that pose unique diagnostic and therapeutic challenges. The present work integrates an updated review of current knowledge on DS-associated ALL with the illustrative presentation of a pediatric case. This patient harbored a rare high-risk genomic profile, a feature rarely reported in DS-ALL, therefore underlining the importance of precise molecular diagnoses and tailored supportive interventions adapted to the complexity of ALL biology and DS-specific vulnerabilities.

### 1.1. Epidemiology and Clinical Background of ALL

ALL is the most common pediatric leukemia, accounting for approximately 75–80% of cases worldwide [1,2,3]. ALL is characterized by the uncontrolled proliferation of immature lymphoid precursors, which invade the bone marrow and can extend to the extramedullary site [4].

Over the past decades, advances in the understanding of ALL biology have resulted in remarkable improvements in diagnosis, treatment, and monitoring [5]. Patient molecular profile, risk-adapted chemotherapy protocols, and enhanced supportive care increased pediatric ALL survival rates to approximately 90% for children younger than 15 years and more than 75% for adolescents aged 15 to 19 [6]. Nevertheless, patients with complex genomic profiles or significant comorbidities face poor prognosis, increased risk of relapse, and inferior clinical outcomes related to high-dose treatment [7,8].

### 1.2. Molecular Diagnostics in Risk Stratification

Advances in molecular diagnostics have influenced the therapeutic decision in ALL. Next-generation sequencing (NGS) and cytogenetic analyses have revealed numerous genetic and epigenetic abnormalities that have prognostic significance [9,10,11]. By integrating these molecular findings with established clinical factors—such as age and presenting white blood cell count (WBC)—clinicians are now able to refine risk stratification and adjust treatment intensity accordingly. This approach seeks to preserve the high-cure rates achieved with intensive therapy, while, at the same time, reducing the likelihood of long-term complications, including secondary malignancies and chronic organ toxicities, which may emerge years after therapy completion [12,13,14].

### 1.3. Down Syndrome as a Distinct High-Risk Subgroup

Children with DS have a significantly higher risk of developing acute leukemia, with a 7- to 20-fold increased incidence compared to the non-DS pediatric population [15,16,17]. DS is characterized by the acquisition of a partial or complete third copy of chromosome 21 [18]. It is the most common single chromosomal disorder in live births, with an incidence of 1 in 300 to 1 in 1100 babies [16,19,20].

The microenvironment established by abnormal hematopoiesis driven by trisomy 21 is compounded by additional genetic and epigenetic changes that can drive leukemogenesis in patients with DS [8].

The diagnostic and therapeutic challenges faced by specific subgroups of patients become increasingly pronounced in children with DS. Epidemiological studies have proved that DS is linked to a high incidence of leukemia in early childhood [21]. Hasle et al. revealed that DS not only predisposes to ALL but also to acute megakaryoblastic leukemia (AML) that occurs at younger ages compared to those without DS [22]. Beyond the immediate concern of a cancer diagnosis, patients with DS often present a cluster of comorbidities, including congenital cardiac abnormalities and immune dysregulation, which can intensify the toxicity of chemotherapy and promote the risk of infections [23,24,25,26,27]. In their review of advancements in DS-ALL, Zwaan et al. emphasized the vital role of effective supportive care and innovative treatment approaches, given the increased morbidity sometimes associated with standard therapeutic regimens [28]. New findings show that improvement in the five-year survival rates, notwithstanding relapse rates and incurable diseases, is still high [29,30]. Patients with DS and ALL tend to have a poorer prognosis than non-DS and ALL due to increased chemotherapy toxicity and higher relapse rates [31].

### 1.4. Molecular Landscape of DS-ALL

Association DS and ALL show a distinctive spectrum of genomic events at the molecular level. One relatively common variant is the Janus kinase 2 (*JAK2*) mutation, often noted as *p.R683G*, which leads to continuous signaling through the JAK-STAT pathway [32,33]. This persistent activation can give leukemic cells a proliferative advantage, which may contribute to drug resistance. Tasian and colleagues investigated the effectiveness of *JAK* inhibitors, namely ruxolitinib, in children with ALL [34]. The preliminary findings suggested that targeted therapies can selectively reduce *JAK2*-mutated clones, but high-dose treatment caused hematological toxicity. Since a lower dosage was often ineffective, ruxolitinib was suspended [35]. Another significant factor is the purinergic receptor *P2Y*, *G-protein-coupled, 8–CRLF2* (*P2RY8-CRLF2*) fusion, which also activates the JAK-STAT pathway and is associated with poorer event-free survival when therapeutic intensification is not risk-based [36].

The gain of chromosome 21 leads to increased expression of chromosome 21 genes and a predisposition to *CRLF2* rearrangements (e.g., *P2RY8–CRLF2*) [37]. Roberts and Mullighan further demonstrated that CRLF2 overexpression often co-occurs with other oncogenic drivers, such as *NRAS* or *KRAS* mutations, thereby enhancing the malignant characteristics of the disease [38]. New research confirmed these findings and highlighted the influence on immune regulation and resistance to therapy [39,40].

A major additional category of genetic contributors to DS-ALL involves abnormalities in the RAS signaling pathway (e.g., mutations in *NRAS* or *KRAS*). Studies by Irving and Harrison revealed that oncogenic *RAS* mutations can sustain the RAS-MAPK pathway, conferring significant resistance to conventional chemotherapies [41]. Building on these findings, Hof et al. proposed that *MEK* inhibitors may act synergistically with standard treatments in children harboring *RAS*-activating mutations [42].

Beyond signaling disruptions, DS-ALL is also characterized by recurrent epigenetic abnormalities, particularly affecting the E1A-binding protein p300 (*EP300*) and *CREBBP* genes, which regulate histone acetylation and transcription [43]. Studies by Tzoneva and Ferrando suggested that pharmacologic modifiers such as histone deacetylase inhibitors could help overcome chemoresistance in epigenetically driven ALL subtypes [44]. However, the safety and efficacy of these targeted or epigenetic therapies require validation in larger clinical trials before incorporation into frontline treatment protocols.

### 1.5. iAMP21 in DS-ALL

Intrachromosomal amplification of chromosome 21 (*iAMP21*) is identified as a high-risk factor in childhood B-precursor ALL [45,46,47]. This abnormality is particularly relevant in children with DS-ALL due to their unique chromosomal context involving chromosome 21. iAMP21 is strongly predictive of poor outcomes and generally necessitates more intensive, MRD-guided therapy [8,45,48,49]. Research from 2014 emphasized the importance of early detection of *iAMP21* through interphase fluorescence in situ hybridization (FISH) to enable timely treatment escalation [48,50]. The flexibility of the FISH probe design has eased rapid detection and disease monitoring in *iAMP21*-positive ALL cases [51]. Moreover, *iAMP21* may cooperate with other genetic lesions in DS-ALL, further underlining the importance of strong supportive care during therapy [49,52].

### 1.6. Minimal Residual Disease (MRD) in DS-ALL Management

In parallel with these molecular advances, the implementation of innovative techniques for MRD monitoring is the strongest independent prognostic predictor in acute leukemia [53,54]. Early studies by van der Velden and van Dongen demonstrated that sufficiently sensitive assays could detect occult leukemic populations below the threshold of traditional morphological detection, enabling earlier adaptive treatment strategies for slow responders [55]. This is particularly essential in DS-ALL, where atypical relapse kinetics and limited options for post-relapse salvage—due to excessive therapy-related toxicity—pose significant challenges.

Large collaborative trials in childhood ALL have incorporated MRD kinetics-based response adaptation [56,57]. They show that MRD assessment enhances the ability to balance treatment escalation against the risk of excessive toxicity [56,57]. By intensifying therapy for clones with high-risk features and de-escalating treatment for leukemia with low-risk characteristics, the overarching aim is to individualize therapy and improve outcomes for each patient.

### 1.7. Multidisciplinary Supportive Care

The complexity of DS-ALL necessitates a comprehensive multidisciplinary approach to care. Due to immune vulnerabilities and a high prevalence of congenital heart disease, patients with DS-ALL often face life-threatening infections and cardiopulmonary complications during treatment [58]. Tandonnet et al. highlighted a significantly increased rate of infection-related mortality in this population, underscoring the urgent need for intensive surveillance, structured antimicrobial prophylaxis, and timely intervention [59]. Collaboration with pediatric cardiologists is vital for carefully regulating anthracycline exposure, as pre-existing cardiac conditions can elevate the risk of anthracycline-induced cardiotoxicity [60]. Furthermore, maintaining ongoing communication with dental specialists is vital to prevent or manage orofacial complications such as mucositis, gingivitis, and invasive dental lesions, which could otherwise impede therapy, as emphasized by Maloney et al. in their supportive care report [61].

### 1.8. Emerging Therapies in DS-ALL

Recently, targeted agents and immunotherapies have been increasingly evaluated to treat DS-ALL. Ruxolitinib and other *JAK* inhibitors have shown promise in specific subgroups harboring *JAK2* alterations, while MEK inhibitors are being explored for *RAS*-driven leukemic blasts. Defective epigenetic regulators such as *EP300* may also be therapeutically targeted with pharmacologic modulators [62]. Immunotherapies, particularly chimeric antigen receptor (*CAR*) T-cell therapy, have achieved favorable responses in relapsed or refractory ALL, although DS-specific clinical data remain limited [63]. Continued refinement of DS-specific biomarkers and treatment strategies holds the potential to produce further improvements in survival outcomes and quality of life [64,65].

### 1.9. Case as a Bridge to the Review

This study describes the case of a three-year-old boy with DS and B-cell ALL (B-ALL) who presented with multiple high-risk genetic abnormalities, including *NRAS*, *JAK2*, and *EP300* mutations; a *P2RY8-CRLF2* fusion; and iAMP21. The unprecedented co-occurrence of these five independent high-risk genomic lesions in a single DS-ALL patient makes this case exceptionally rare. It provides a rare opportunity to discuss the implications for MRD surveillance and anticipatory supportive care.

The case highlights how genomic profiling, MRD-guided treatment, and multidisciplinary supportive care—including infection prophylaxis, cardioprotective measures, and structured dental management—can optimize outcomes in DS-ALL.

Although ALL remains the most common childhood malignancy, with overall survival rates exceeding 80%, children with DS represent a unique subgroup, characterized by an increased risk of leukemia, distinctive molecular alterations, and increased treatment-related morbidity and mortality. Lesions such as *JAK2* mutations, *CRLF2* rearrangements, and RAS pathway alterations are previously described in DS-ALL [66,67,68,69,70], but the simultaneous presence of multiple high-risk genomic abnormalities is exceedingly rare. Such cases help understand disease biology, mechanisms of treatment resistance, and the need for tailored supportive care.

While previous studies have evaluated the prognostic significance of individual genetic drivers in DS-ALL [71], few have examined the clinical implications of their co-occurrence or integrated them with supportive care challenges—including infection risk, cardiotoxicity, and oral/dental complications. This gap hampers the development of risk-adapted, multidisciplinary strategies for the DS-ALL group.

The objective of this study is to report the first documented pediatric DS-ALL case harboring five concurrent high-risk genomic lesions (*NRAS p.G13D*, *JAK2 p.R683G*, EP300 p.Q1766*, *P2RY8–CRLF2* fusion, and *iAMP21*). The first aim of this study is to illustrate the patient’s clinical presentation, including the genomic profile, treatment course, and supportive care needs, and, second, to place these findings in the context of the existing literature, emphasizing relapse and complications.

## 2. Materials and Methods

This work combines a pediatric DS-ALL case report with a narrative literature review to integrate the clinical and genomic findings within the current knowledge of therapy and chemotherapy-related complications in DS-ALL.

### 2.1. Literature Review Strategy

The PubMed search (January 2020–June 2025) using the terms ‘Down syndrome’ and ‘acute lymphoblastic leukemia’ retrieved 93 publications, of which 17 were reviews and 10 were case reports. Additional targeted searches combining ‘Down syndrome’ and ‘acute lymphoblastic leukemia’ with specific high-risk genomic lesions (*JAK2*, *NRAS*, *CRLF2*, *EP300*, and *iAMP21*) yielded no publications describing all five alterations in a single DS-ALL patient.

Inclusion criteria included peer-reviewed studies, clinical cohorts, case series, and case reports that described DS-ALL patients with defined genomic lesions. Exclusion criteria included non-English publications and reviews or editorials lacking primary information.

### 2.2. Diagnostic, Immunophenotypic, and Genomic Procedures

*Cytogenetics*: Conventional karyotyping and chromosomal analysis were completed on peripheral blood lymphocytes cultured under standard conditions, using G-banding at a resolution of 400–550 bands per haploid set. Both metaphase analysis and karyotype interpretation followed the International System for Human Cytogenomic Nomenclature (ISCN 2020).

*Bone marrow and flow cytometry*: Bone marrow aspirates were collected from the posterior iliac crest under sterile conditions and sedation. Samples were processed according to laboratory protocols, and immunophenotyping was performed by multiparameter flow cytometry using an antibody panel including CD19, CD34, CD10, CD58, and additional markers relevant for B-cell precursor ALL.

*Minimal residual disease (MRD)*: MRD assessment was carried out at day 15 and day 33 of induction therapy in accordance with departmental ALL/ALL-BFM guidance, using eight-color multiparameter flow cytometry (including CD19, CD10, CD34, and CD58). The validated limit of detection was 10^−4^ (0.01%).

*Fluorescence in situ hybridization (FISH)*: FISH assays targeting *iAMP21* were performed using manufacturer-validated probes, following standard laboratory operating procedures with internal and external controls for quality assurance.

*Extended molecular profiling (NGS)*: Genomic DNA and total RNA were extracted from bone marrow aspirates using the QIAamp DNA Mini Kit and RNeasy Mini Kit (Qiagen, Hilden, Germany), according to the manufacturer’s instructions. Library preparation was conducted with a custom targeted amplicon leukemia panel optimized for pediatric B-ALL (54 genes), including point mutations (*JAK2*, *NRAS*, *KRAS*, *TP53*, *EP300*, *CREBBP*, *IKZF1*, *CDKN2A*, and *PAX5*) and recurrent fusions (*P2RY8-CRLF2*, *ETV6-RUNX1*, *BCR-ABL1*, *KMT2A* rearrangements, and *TCF3-PBX1*). Sequencing was performed on an Illumina MiSeq platform (paired-end 2 × 150 bp), achieving ≥500× mean coverage depth for DNA libraries and ≥2 million reads for RNA libraries. Reads were aligned to GRCh38/hg38; variant calling was performed with *GATK* v4.0 and fusion detection with STAR-Fusion. Reporting thresholds were set at variant allele frequency (VAF) ≥5% and ≥10 uniquely aligned reads for fusions. Variant interpretation followed the ACMG/AMP 2015 guidelines for somatic variants in cancer.

### 2.3. Ethical Considerations

This study complied with the Declaration of Helsinki. The Institutional Review Board waived the requirement for formal approval, as this non-interventional case report met the exemption criteria. Written informed consent for participation and publication of anonymized clinical and genomic data, including clinical images, was obtained from the patient’s parents.

### 2.4. Statistical Considerations

Given the single-patient design, no inferential statistics were performed. Quantitative data are presented descriptively (laboratory values, percentages, and time points). This study is hypothesis-generating and does not permit causal inference.

## 3. Results

### 3.1. Clinical Presentation and Baseline Characteristics

A three-year-old boy with DS (non-mosaic complete trisomy 21) and a congenital heart defect (patent ductus arteriosus, PDA) was admitted to the Pediatric Hematology Unit at Fundeni Clinical Institute with clinical signs suggestive of acute leukemia. Written informed consent for diagnostic and therapeutic procedures was obtained from the patient’s legal guardians.

The patient was the first child of healthy, non-consanguineous parents (maternal age 35, paternal age 36), with no family history of hematologic, oncologic, or genetic disorders. He was born at term with appropriate weight and Apgar scores of 10 at one and five minutes. Cytogenetic analysis at birth confirmed free de novo trisomy 21, most likely resulting from maternal meiotic nondisjunction in the context of advanced maternal age.

Patient examination on admission revealed pallor, petechiae, cervical lymphadenopathy, and hepatomegaly. Laboratory testing showed leukocytosis (26.08 × 10^9^/L), mild anemia (hemoglobin 10.9 g/dL), thrombocytopenia (81 × 10^9^/L), elevated lactate dehydrogenase (458 U/L), and C-reactive protein (49 mg/L). Peripheral smear revealed approximately 54% blasts. Bone marrow aspirate showed ~95% lymphoblasts among nucleated cells, with a B-cell precursor immunophenotype (CD19^+^, CD34^+^, and CD58^+^). Baseline clinical and laboratory findings are summarized in Table 1.

### 3.2. Genomic Profile

Targeted amplicon-based next-generation sequencing (54-gene pediatric ALL panel) together with cytogenetic analysis of the bone marrow aspirate identified five independent high-risk genomic lesions: *NRAS c.38G > A (p.G13D)*, *JAK2 c.2047C > G (p.R683G)*, EP300 c.5296C > T (p.Q1766*), *P2RY8–CRLF2* fusion, and *iAMP21* (Table 2). The tumor mutational burden was low, and no microsatellite instability was detected. Although these genomic abnormalities did not necessitate modifications to the induction regimen, they justified closer MRD surveillance and the adoption of intensified supportive care measures.

### 3.3. Induction Therapy and Early Treatment Response

The patient commenced multi-agent induction chemotherapy (Vincristine, Doxorubicin, PEG-Asparaginase, and Corticosteroids) with cardiology-guided dose adjustments due to PDA.

- Day 15: MRD ~1.8%, indicating partial cytoreduction.
- Day 33: Complete morphological remission with undetectable MRD; platelet recovery to 221 × 10^9^/L; hemoglobin stable at 9.7 g/dL.

Hematologic evolution during induction is presented in Table 3.

**Figure 1:**
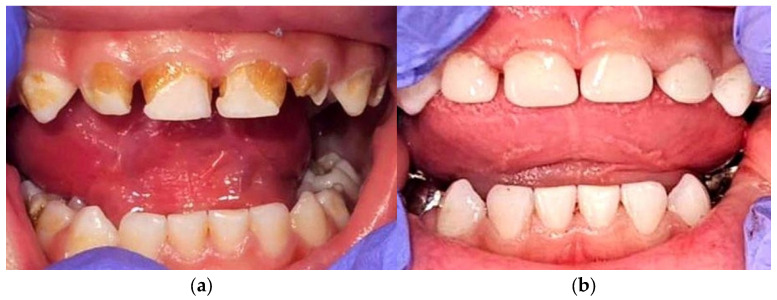
Dental complications and restorative management in DS-ALL. (**a**) Intraoral presentation after induction chemotherapy, showing extensive structural and chromatic alterations of the deciduous dentition, including pronounced enamel hypoplasia, generalized discoloration, and multiple carious lesions, in the context of DS and prior systemic chemotherapy; (**b**) post-treatment restorative reconstruction of the affected teeth using composite resin fillings and prefabricated pediatric crowns, aimed at restoring function and aesthetics, while minimizing the risk of odontogenic infections. Abbreviations: DS—Down syndrome; ALL—acute lymphoblastic leukemia.

To facilitate a comprehensive understanding of the temporal sequence of key clinical, genomic, and therapeutic events, we provide in Figure 1 a schematic time-course representation summarizing the patient’s diagnostic, therapeutic, and supportive care milestones.

### 3.4. Infectious Complications and Supportive Measures

Two major bacterial infections occurred during induction:

- *Klebsiella pneumoniae* bloodstream infection, managed with broad-spectrum antibiotics tailored to sensitivity testing.
- *Pseudomonas fluorescens* orchitis, required escalated targeted therapy.

Supportive measures included tranexamic acid, fluconazole prophylaxis, acyclovir, fluid/electrolyte management, and protective isolation, which prevented sepsis progression and avoided treatment delays.

### 3.5. Dental Complications and Interdisciplinary Care

Post-induction, the patient developed chemotherapy dental pathology: enamel hypoplasia, discoloration, and multiple carious lesions (Figure 1a). Restorative management included composite fillings and pediatric crowns (Figure 1b). Preventive dental monitoring was maintained to minimize infection risk during subsequent therapy.

**Figure 2:**
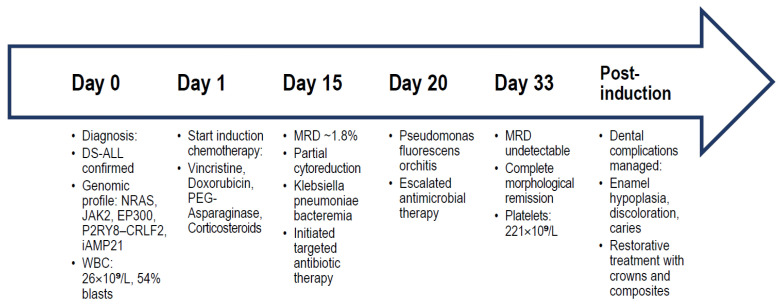
Timeline summarizing the patient’s diagnostic work-up, genomic findings, induction chemotherapy course with MRD assessments, infectious complications, and dental management. The diagram integrates (i) initial diagnosis and baseline laboratory values, (ii) genomic and cytogenetic alterations identified by NGS and karyotyping, (iii) induction chemotherapy timeline with MRD assessments, (iv) infectious complications and their targeted antimicrobial management, and (v) dental pathology development and restorative interventions. Abbreviations: DS—Down syndrome; ALL—acute lymphoblastic leukemia; WBC—white blood cell count; NGS—next-generation sequencing; MRD—minimal residual disease; PDA—patent ductus arteriosus.

To illustrate the patient’s clinical course, genomic profile, treatment milestones, and major complications in an integrated manner, a time-course diagram was constructed (Figure 2). The schematic depicts the chronological sequence from initial diagnosis to the completion of induction therapy, emphasizing diagnostic, therapeutic, infectious, and dental complications.

### 3.6. Case Report Highlights

- To our knowledge, the first pediatric DS-ALL case documented with five concurrent high-risk genomic lesions (*NRAS p.G13D*, *JAK2 p.R683G*, EP300 p.Q1766*, *P2RY8*–CRLF2 fusion, and *iAMP21*).
- Early MRD-negative remission was achieved by Day 33 despite an aggressive genomic profile.
- Severe infectious complications (*Klebsiella pneumoniae* bacteremia and *Pseudomonas fluorescens* orchitis) were successfully managed with targeted antimicrobials and protective isolation.
- Chemotherapy-exacerbated dental pathology (enamel hypoplasia, discoloration, and caries) reported and managed with restorative interventions—first DS-ALL case to detail this complication.
- Multidisciplinary care (cardiology-guided chemotherapy, structured dental interventions, and DS-specific infection prophylaxis) ensured treatment continuity and reduced morbidity.

## 4. Discussion

This case highlights the clinical and molecular complexity of DS-ALL, particularly in the presence of multiple concurrent high-risk alterations (including *JAK2*, *NRAS*, and *EP300* mutations, the *P2RY8-CRLF2* fusion, and *iAMP21*). Despite this profile, early MRD-negative remission was achieved through coordinated, risk-adapted chemotherapy, guided by genomic profile, and supported by multidisciplinary care.

### 4.1. Integration of Previous Genomic and Clinical Studies (2007–2025)

Many recent studies have described individual genomic alterations in DS-ALL, but such a combination of high-risk alterations has never been reported in a single patient. Hurtado et al. (2023) [72] reviewed the role of *JAK2* in Ph-like B-ALL. They reported that *JAK2* mutations and rearrangements are present in ~3.5% of all B-ALL cases but in nearly 19% of DS-ALL cases, confirming their enrichment in this subgroup and association with poor prognosis [72].

Earlier reports provided direct clinical evidence: Malinge et al. (2007) described a novel activating *JAK2* mutation in a DS patient with B-cell precursor ALL, while Kearney et al. (2009) documented the recurrent *JAK2 R683* mutation together with multiple gene deletions [66,67]. Hertzberg et al. (2010), analyzing samples from the International BFM Study Group, demonstrated that *JAK2* mutations are linked to aberrant CRLF2 expression in DS-ALL, establishing the *JAK2*–CRLF2 axis as a cooperative driver of leukemogenesis [68].

*CRLF2*-driven leukemogenesis itself has been further investigated in recent studies. Page et al. (2022) identified HMGN1, a chromosome 21-encoded gene, as a significant enhancer of *CRLF2*-mediated leukemic transformation, showing a chromosome 21 dosage effect in DS-ALL [69]. Balestra et al. (2025) proved that targeting the thymic stromal lymphopoietin receptor (TSLPR) reduced resistance to immunotherapy in *CRLF2*-rearranged and DS-ALL, offering potential for therapeutic interventions [70].

Regarding the RAS pathway, Koschut et al. (2021) demonstrated that activation of RAS signaling—not merely the mutational status—was a predictor of outcome in high-risk ALL [73]. They proposed *RAS* activation as a unifying therapeutic vulnerability, particularly relevant in DS-ALL, where *NRAS* and *KRAS* mutations are frequent [73].

To our knowledge, this represents the first report of an *EP300* mutation in DS-ALL, extending previous observations in pediatric ALL more broadly to the DS-associated context. While no studies to date have documented EP300 mutations specifically in DS-ALL, epigenetic regulators such as *EP300* and *CREBBP* are recurrently altered in pediatric ALL and have been implicated in treatment resistance and transcriptional dysregulation. A recent review by Peroni et al. (2023) confirms the role of epigenetic disruption in leukemogenic predisposition and the broader cellular and molecular heterogeneity of hematologic malignancies in DS [74]. These malignancies are characterized by a combination of chromosomal abnormalities, oncogenic mutations, and epigenetic deregulation [74].

Regarding *iAMP21*, Verdoni et al. (2022) reported a rare case of DS-ALL with *iAMP21* due to a constitutional isodicentric chromosome 21, showing that this high-risk cytogenetic lesion confers an adverse prognosis [52].

Roberts (2022) further highlighted the markedly increased risk of both AML and ALL in young children with DS, framing the unique developmental susceptibility of this population [75]. Together, these studies show that individual high-risk lesions—including *JAK2* mutations, *CRLF2* fusions, *RAS* activation, or *iAMP21*—have been separately described in DS-ALL. However, the simultaneous occurrence of all five alterations, as reported in the present case (*JAK2 R683G*, *NRAS G13D*, EP300 Q1766*, *P2RY8–CRLF2* fusion, and *iAMP21*), has not been documented previously.

These findings point to the molecular heterogeneity of DS-ALL and the importance of systematic genomic profiling for accurate risk assessment. Building upon this context, our case further illustrates how early, risk-adapted intensification and supportive care can overcome the challenges posed by an exceptionally adverse genomic background.

### 4.2. NRAS p.G13D in the Context of DS-ALL and Other Disorders

Regarding the specific *NRAS p.G13D* variant observed in our patient, this mutation has also been described in the past five years across distinct hematologic and non-hematologic disorders. Beyond DS-ALL, *NRAS p.G13D* has been recurrently described in RARA-negative acute promyelocytic-like leukemia with concomitant myelodysplastic syndrome [76], in lupus nephritis through aberrant RAS-MAPK signaling [77], in colorectal cancer [78], and in histiocytosis syndromes [79]. These findings underscore that *NRAS p.G13D* is a recurrent driver mutation with pleiotropic pathogenic effects, whose biological impact depends on the genomic and epigenetic context. In DS-ALL, its co-occurrence with *JAK2 R683, CRLF2* rearrangement, *EP300* loss-of-function, and *iAMP21* suggests synergistic pathway of deregulation and likely accounts for the aggressive molecular phenotype observed in our patient.

### 4.3. Early Intensification and Supportive Care

Although the chemotherapy protocol adhered to standard treatment guidelines, therapeutic management was individualized according to the patient’s genomic profile. This involved coordination of an interdisciplinary team and risk-adapted decision-making. Despite the presence of multiple high-risk molecular alterations, the patient achieved early remission by Day 33. At the same time, the occurrence of severe infections —namely, *Klebsiella pneumoniae* bacteremia and a *Pseudomonas fluorescens*-associated testicular lesion—underscores the heightened vulnerability to life-threatening infections in DS, likely a consequence of inherent immune dysregulation and associated comorbidities [8,80,81]. Intensive infection surveillance and targeted, preemptive antibiotic therapy, initiated at the earliest signs of febrile illness or focal symptoms, were instrumental in enabling continuous chemotherapy delivery, which is essential for maintaining leukemic control.

At the time of manuscript submission, the patient remained in complete remission following induction therapy. However, we acknowledge the limitation of not yet having long-term follow-up data, which remains pending.

The induction phase of ALL therapy remains the most important for achieving remission and the period most fraught with risk for life-threatening complications. Successful induction therapy in DS-ALL depends equally on achieving cytoreduction and on providing anticipatory supportive care with prompt recognition and management of severe toxicities [82].

### 4.4. Management of Comorbidities

The presence of a congenital cardiac defect is an additional factor of complexity, necessitating frequent cardiac monitoring and potential adjustments of anthracycline dose to reduce the risk of cardiotoxicity [83]. In addition, regular dental consultations and prophylactic dental care were incorporated into the supportive care plan to prevent oral mucositis, gingival infections, and invasive dental lesions [84].

Although no central nervous system (CNS) involvement was detected at diagnosis, prophylactic intrathecal chemotherapy remained a vital element of the treatment strategy, given the recognized risk of extramedullary spread in this population [85,86].

Another important clinical consideration involves chemotherapy-related oral complications, which are particularly relevant in children with DS due to their inherent oromaxillofacial vulnerabilities.

During the induction and later treatment phases, the patient developed mucosal sensitivity and enamel demineralization, complications associated with immunosuppression and xerostomia, both known effects of intensive chemotherapy [84,87,88]. The lower anterior teeth were less affected, likely because of protection from sublingual and submandibular salivary secretions. Preventive dental care and early involvement of pediatric dental specialists helped reduce the risk of oral infection, discomfort, and nutritional difficulties during therapy [89].

As shown in the clinical photographs (Figure 1), the patient had characteristic dysmorphic features and developed clear oral changes during chemotherapy, including mucositis of the non-keratinized mucosa, marked discoloration, and enamel hypoplasia.

These findings underline the need for continuous dental monitoring and prophylaxis in children with DS-ALL receiving intensive chemotherapy. The oral manifestations observed during treatment reflect the broader systemic burden of cytotoxic therapy.

Children with DS are predisposed to periodontal disease, delayed dental eruption, enamel hypoplasia, and immune dysfunction—all factors that heighten the risk of dental complications during cancer treatment [90,91]. The combined effects of chemotherapy-induced mucositis, neutropenia, and compromised oral hygiene can lead to secondary infections, impaired nutrition, and decreased quality of life [92]. Notably, children who undergo chemotherapy before the age of five appear to be at higher risk for more severe long-term dental effects [93,94].

Given these risks, multidisciplinary management involving pediatric oncologists, dentists, and nutritionists is imperative from the early phases of diagnosis. Prophylactic dental care, rigorous oral hygiene protocols, and preventive agents such as fluoride and chlorhexidine may reduce complications [95,96,97]. Future protocols for DS-ALL should consider integrating structured dental assessments into supportive care guidelines, recognizing the oral cavity as both a potential site of infection and a reflection of systemic tolerance to therapy.

### 4.5. Molecular Targets and Future Therapies

At the molecular level, the concurrent activation of the JAK-STAT and RAS-MAPK pathways underscores the potential of targeted therapies—such as *JAK* or MEK inhibitors—as adjunctive or salvage options in cases where standard therapy is ineffective. Similarly, the *EP300* mutation, indicative of epigenetic dysregulation, supports the rationale for exploring histone deacetylase inhibitors or other epigenetic agents, especially in cases of persistent MRD.

Nevertheless, these precision therapies must be balanced against the increased infection risk and overlapping toxicities to which children with DS are particularly vulnerable. The link between genomic vulnerability and phenotypic expression in DS-ALL strengthens the importance of personalized therapeutic strategies that address both oncologic management and supportive care. In addition to achieving disease control, these strategies should prioritize the preservation of long-term functional outcomes and overall quality of life.

### 4.6. Limitations

This study has several limitations that must be acknowledged. First, it describes a single pediatric case, which restricts the generalizability of our findings. Although the co-occurrence of five independent high-risk genomic lesions is unprecedented, our conclusions should be regarded as hypothesis-generating rather than definitive. Secondly, long-term follow-up data are not yet available, so the durability of remission, the risk of relapse, and late toxicities cannot be assessed at this stage. Thirdly, although genomic profiling was extensive, functional validation of the identified mutations was not performed, and the therapeutic significance of these alterations remains inferred from previous studies. Finally, the literature review, although structured, may be subject to selection bias due to its focus on English-language publications and the heterogeneity of reports available. Despite its limitations, this case illustrates the potential value of combining detailed molecular diagnostics with personalized supportive care in children with DS-ALL. It suggests the need for larger multicenter studies to confirm and expand these observations.

### 4.7. Outcomes of Current Protocols in DS-ALL

In Table 4 we summarize the key findings from the current literature about outcome of DS-ALL therapy in children.

The management of high-risk B-ALL in children with DS generally follows the same protocols used for children without DS; however, specific adjustments and closer monitoring are required because of comorbidities and increased treatment-related toxicity.

Recent reports indicate improved outcomes in DS-ALL when modified chemotherapy regimens are applied [24,101]. Event-free survival and overall survival have risen compared with earlier clinical trials but remain lower than in non-DS ALL at five years, with relapse still occurring more often in the DS group [24,98]. These improvements are mainly linked to the introduction of less toxic, DS-adapted treatment protocols and advances in supportive care, which together have lowered treatment-related mortality and improved tolerability [24,101].

Despite these gains, a large retrospective study with long-term follow-up ranging from 5 to 10 years, including 1303 cases of DS-ALL and 30,173 cases of non-DS-ALL, reported comparable treatment outcomes between the two groups; however, treatment-related mortality was significantly higher in the DS cohort [102].

Regarding novel therapies, Sora et al. [103] described three cases of relapsed/refractory DS-ALL treated with blinatumomab. All the patients achieved remission after a single treatment cycle, with no significant toxicity, suggesting a potential role for targeted immunotherapy in this high-risk subgroup.

### 4.8. Key Findings

This case shows that remission can be achieved despite an adverse molecular profile when treatment is timely, targeted, and individualized. Both precision medicine and supportive care are essential components in the management of DS-ALL.

Multidisciplinary coordination facilitates the safe delivery of intensified chemotherapy, while systematic oral health monitoring should be recognized as an essential component of supportive care.

## 5. Suggestions for Future Research

Future research should focus on:

- Advanced molecular profiling to define risk stratification and support earlier integration of targeted therapies;
- Evaluation of integrated care models to assess the impact of multidisciplinary coordination on survival and treatment tolerance;
- Prospective studies on oral health interventions aimed at reducing complications and improving the quality of life in patients with DS-ALL.

## 6. Conclusions

This report documents, to our knowledge, the first pediatric DS-ALL case harboring five concurrent high-risk genomic lesions. Despite this exceptionally adverse molecular profile, the patient achieved early MRD-negative remission through risk-adapted chemotherapy supported by multidisciplinary care. The findings emphasize the importance of integrating molecular diagnostics with individualized supportive measures, particularly in infection control, cardiology, and oral health.

Notably, the case illustrates that precision-based supportive care is as critical as oncologic therapy in DS-ALL. Although limited by the one-case design and short follow-up, this report provides a basis for hypothesis generation and may inform the design of future multicenter studies. A deeper understanding of how complex genomic features interact with DS-specific vulnerabilities could guide the development of safer, more effective, and patient-centered DS-ALL therapeutic strategies.

## Figures and Tables

**Table 1:** Baseline parameters at diagnosis.

Parameter	Value	Normal Range	Interpretation
WBC (×10^9^/L)	26.08	5.0–15.0	Leukocytosis
Blasts in smear (%)	~54%	0%	Marker of acute leukemia
Hemoglobin (g/dL)	10.9	11.5–13.5	Mild anemia
PLT (×10^9^/L)	81	150–450	Thrombocytopenia
CRP (mg/L)	49	<5	Inflammatory response
LDH (U/L)	~458 (elevated)	<250	Accelerated cellular turnover
Clinical Features	Lymphadenopathy, petechiae, DS facies, PDA	-	Systemic infiltrative disease

WBC—white blood cell count; CRP—C-reactive protein; LDH—lactate dehydrogenase; PDA—patent ductus arteriosus; PLT—platelet count; DS—Down syndrome.

**Table 2:** High-risk genomic alterations.

Gene/Abnormality	Alteration Type	Clinical Significance
*NRAS p.G13D*	Point mutation	RAS–MAPK activation; relapse risk, chemoresistance
*JAK2 p.R683G*	Point mutation	JAK–STAT hyperactivation; frequent in DS-ALL
EP300 p.Q1766*	Truncating mutation	Epigenetic disruption; potential HDAC inhibitor target
*P2RY8-CRLF2*	Gene fusion	CRLF2 overexpression; variable prognosis
*iAMP21*	Amplification	High-risk cytogenetic lesion, potential negative prognostic marker

ALL—acute lymphoblastic leukemia; DS—Down syndrome; HDAC—histone deacetylase; *iAMP21*—intrachromosomal amplification of chromosome 21.

**Table 3:** Evolution of hematological parameters and major complications.

Parameter	Day 15	Day 33	Interpretation
MRD (%)	~1.8	Undetectable	Deep remission
Hemoglobin (g/dL)	8.5	9.7	Moderate fluctuations; RBC transfusion during aplasia
WBC (/mm^3^)	1.500	3.970	Pronounced neutropenia during aplasia
PLT (×10^9^/L)	20	221	Significant increase after critical aplasia phase
Nosocomial Infections	*Klebsiella pneumoniae*, *Pseudomonas fluorescens*	None	Managed with broad-spectrum antibiotic therapy
Hemorrhagic Manifestations	Mild	Absent	Prophylaxis with tranexamic acid
Clinical Status	Stable	Clinical remission	No meningeal signs, good general status at discharge

MRD—measurable residual disease; WBC—white blood cell count; PLT—platelet count; RBC—red blood cell.

**Table 4:** Summary of findings in the recent literature about outcome of treatment of DS-ALL.

Study Type and Year	Therapy	Patients	Results	Complications
Retrospective observational study 2021 [31]	Not specified.	14 DS-ALL patients vs. ALL controls	Survival in DS-ALL: 35.7%; in non-DS ALL: 75.8%. Relapse rate higher in DS-ALL.	Chemotherapy-related infections and toxicities
Match cohort study 2021 [98]	Not specified.	251 subjects	MRD at end of induction similar between DS and non-DS groups. Higher relapse risk in DS-ALL.	Not specified.
Case report 2023 [99]	Standard chemotherapy	2-year-old boy	Complete remission; later relapses in bone marrow and CNS.	Recurrence (twice)
Case report 2025 [100]	Sequential therapies: reduced-dose MTX and prednisolone → ALL-REZ BFM-2002 → Inotuzumab-based salvage regimens	Male, diagnosed at age 3	Initial remission; relapse at 15 months; second remission after 20 months of inotuzumab-based therapy.	Persistent fever, joint pain, prolonged thrombocytopenia
Case report 2025 [86]	VDLP regimen + CNS prophylaxis (intrathecal chemo via lumbar puncture)	11-year-old male	MRD-negative status achieved.	Acute paraplegia, progressive lower back pain

DS—Down syndrome; ALL—acute lymphoblastic leukemia; MRD—minimal residual disease; VDLP—Vincristine, Daunorubicin, L-asparaginase, Prednisone; MTX—Methotrexate; CNS—central nervous system.

## Data Availability

Data are available from the corresponding author upon reasonable request.

## Abbreviations

The following abbreviations are used in this manuscript:

ALL	acute lymphoblastic leukemia
DS	Down syndrome
*JAK2*	Janus kinase 2
*NRAS*	neuroblastoma RAS viral oncogene homolog
*EP300*	E1A-binding protein p300
*CRLF2*	cytokine receptor-like factor 2
*iAMP21*	intrachromosomal amplification of chromosome 21
MRD	minimal residual disease
B-ALL	B-cell ALL
NGS	Next-generation sequencing
AML	megakaryoblastic leukemia
*P2RY8-CRLF2*	receptor P2Y, G protein-coupled, 8–CRLF2
FISH	fluorescence in situ hybridization
WBC	white blood cell count
Hb	hemoglobin
CRP	C-reactive protein
LDH	lactate dehydrogenase
PDA	patent ductus arteriosus
PLT	platelet count
HDAC	histone deacetylase
TSLPR	thymic stromal lymphopoietin receptor
VDLP	Vincristine, Daunorubicin, L-asparaginase, Prednisone
MTX	Methotrexate
CNS	Central Nervous System
